# Supplementary material for: Usb1 controls U6 snRNP assembly through evolutionarily divergent cyclic phosphodiesterase activities
Source: Nat Commun. 2017 Sep 8;8:497. doi: 10.1038/s41467-017-00484-w (PMC5591277; doi:10.1038/s41467-017-00484-w)
Supplement: Supplementary file 1 — Supplementary Information [file 41467_2017_484_MOESM1_ESM.pdf]

Description of Supplementary Files

File name: Supplementary Information
Description: Supplementary figures, supplementary tables and supplementary references.

File name: Peer review file

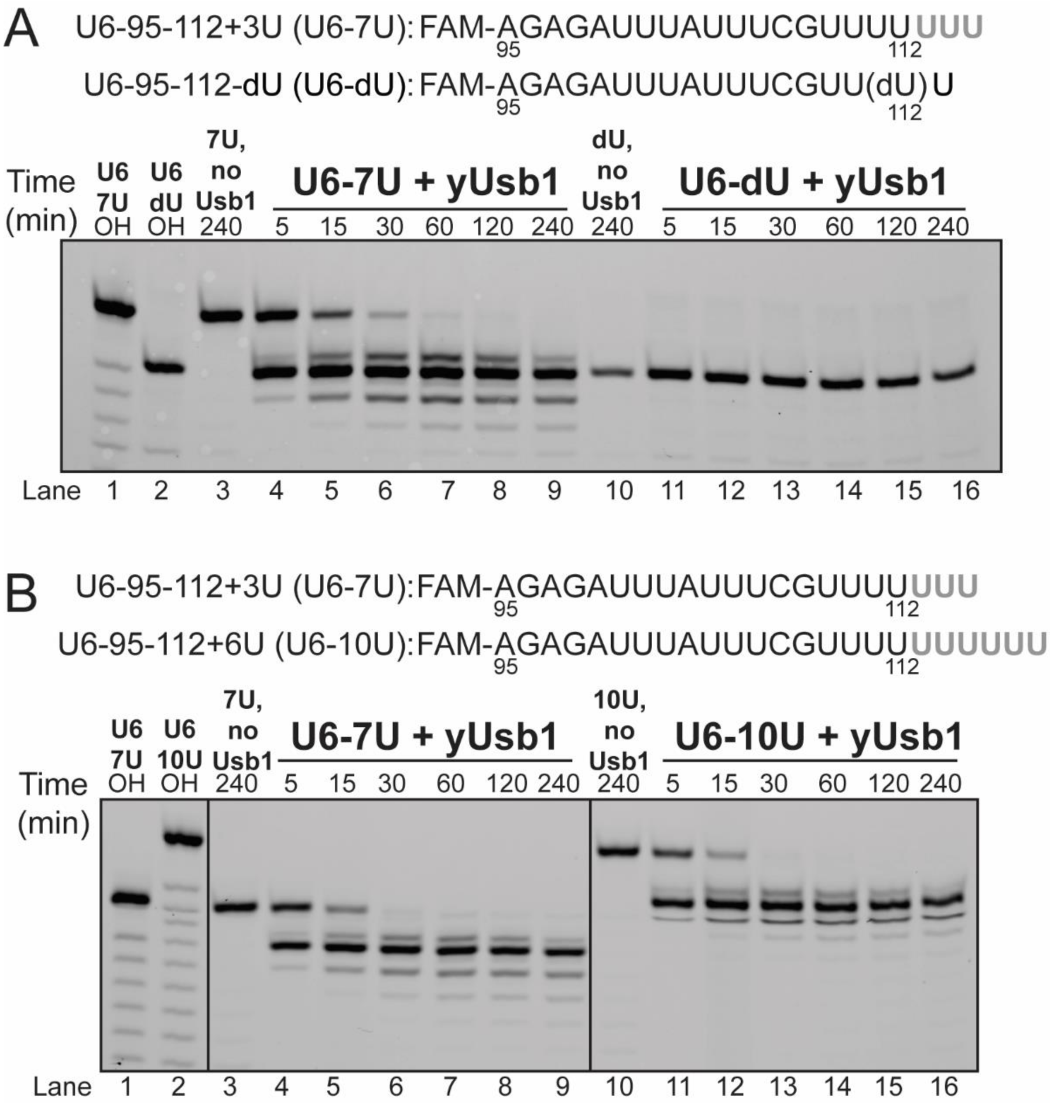

Supplementary Figure 1. Usb1 is inactive on substrates with a deoxyuridine adjacent to the scissile phosphate.

(a) Time course of Usb1 processing on substrates with three additional uridines (U6-7U, lanes 3-9) or where the *n-1* nucleotide is a deoxyuridine (U6-dU, lanes 10-16) indicate that yUsb1 processing is exonucleolytic and depends on a 2' hydroxyl. Alkaline hydrolysis ladders of the RNAs (lanes 1-2) show the mobility of oligonucleotide products of different length. (b) Time course of Usb1 processing on substrates with three additional uridines (U6-7U, lanes 3-9) or six additional uridines (U6-10U, lanes 10-16) indicate that yUsb1 processing is independent of U-tail length. Alkaline hydrolysis ladders of the RNAs (lanes 1-2) show the mobility of oligonucleotide products of different length.

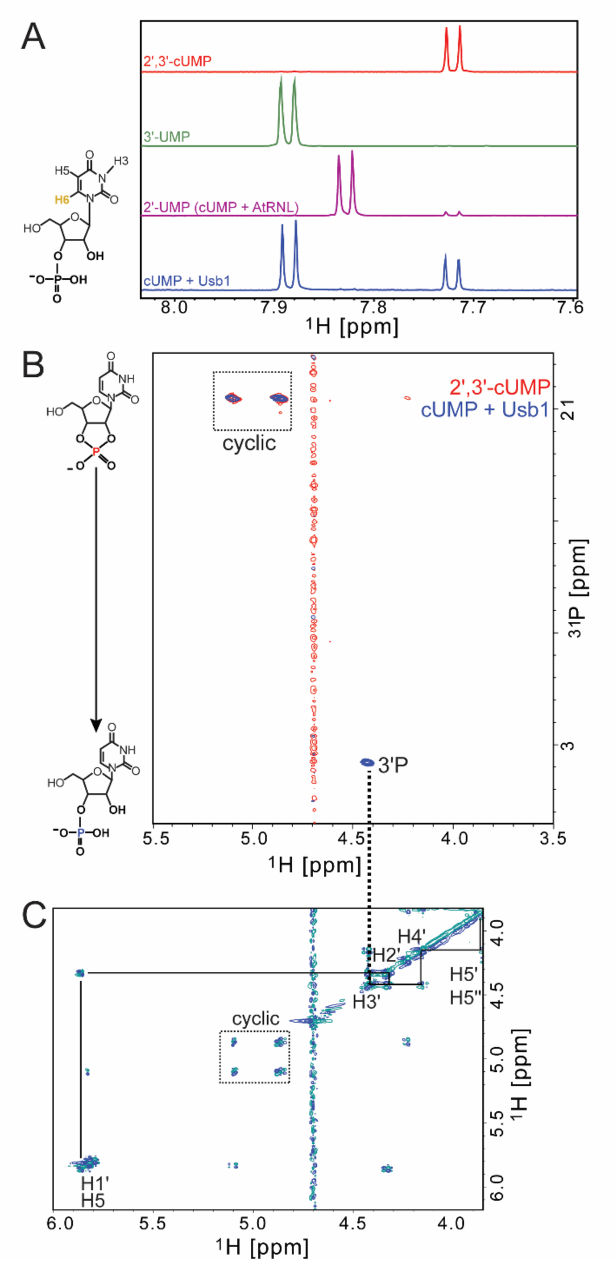

Supplementary Figure 2. NMR of Usb1-treated 2',3'-cUMP reveals Usb1 has CPDase activity and leaves a 3' phosphate.

(a) 1D-^{1}H spectra focusing on the ^{1}H chemical shift region for uracil H6 protons. 2',3'-cyclic UMP (red) was incubated with AtRNL (purple) or Usb1 (blue). The spectra of a 3' UMP standard is also shown. The H6 proton peaks are split by proton-proton coupling between H5 and H6. (b) 2D-^{1}H-^{31}P HMBC overlay of 2',3'-cUMP before (red) and after incubation with AtRNL (purple) or Usb1 (blue). (c) 2D-^{1}H-^{1}H COSY of 2',3'-cUMP after incubation with Usb1.

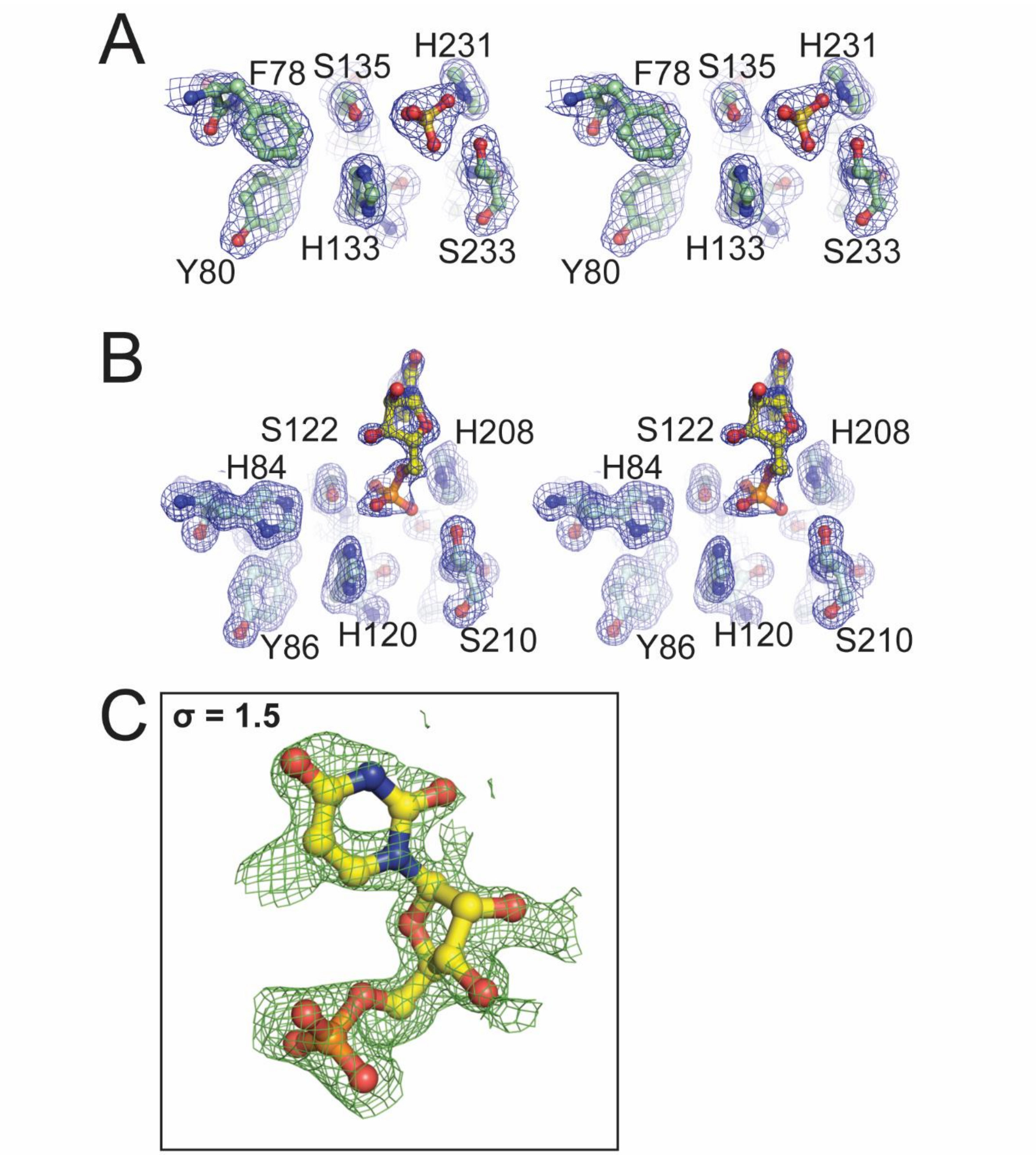

Supplementary Figure 3. Active site densities and annealed omit map.
(a) Stereo view of the $2mF_o$-DF_c electron density map of the yUsb1 active site, contoured at 1σ. (b) Stereo view of the $2mF_o$-DF_c electron density map of the hUsb1/5'UMP active site, contoured at 1σ. (c) Annealed omit map of the hUsb1/5'UMP active site. The occupancy of the bound ligand was refined to a value of 0.72 in phenix.refine. The residual F_o-F_c density near to the C5 atom overlaps with a bound water molecule in the ligand-free structure reported previously[1] (PDB 4H7W).

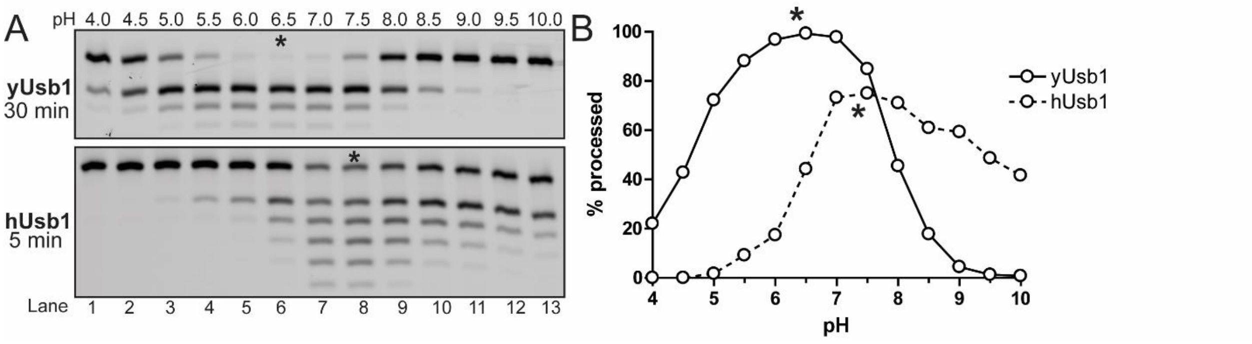

Supplementary Figure 4. yUsb1 and hUsb1 have different pH optimums.

(a) yUsb1 and hUsb1 have different pH optimums (indicated by an asterisk) and different tolerances for low and high pH. Single time-point comparison of yUsb1 (top; 30 minute time point) and hUsb1 (bottom; 5 minute time point) in conditions with pH ranging from 4.0 - 10.0. yUsb1 is most active at pH ~6.5 and is active over a range from pH 4.0 - 8.5. hUsb1 is most active at pH ~7.5 and is active over a range from pH 5.5 - 10.0. (b) Quantification of (a) showing the amount of substrate depleted as a function of pH.

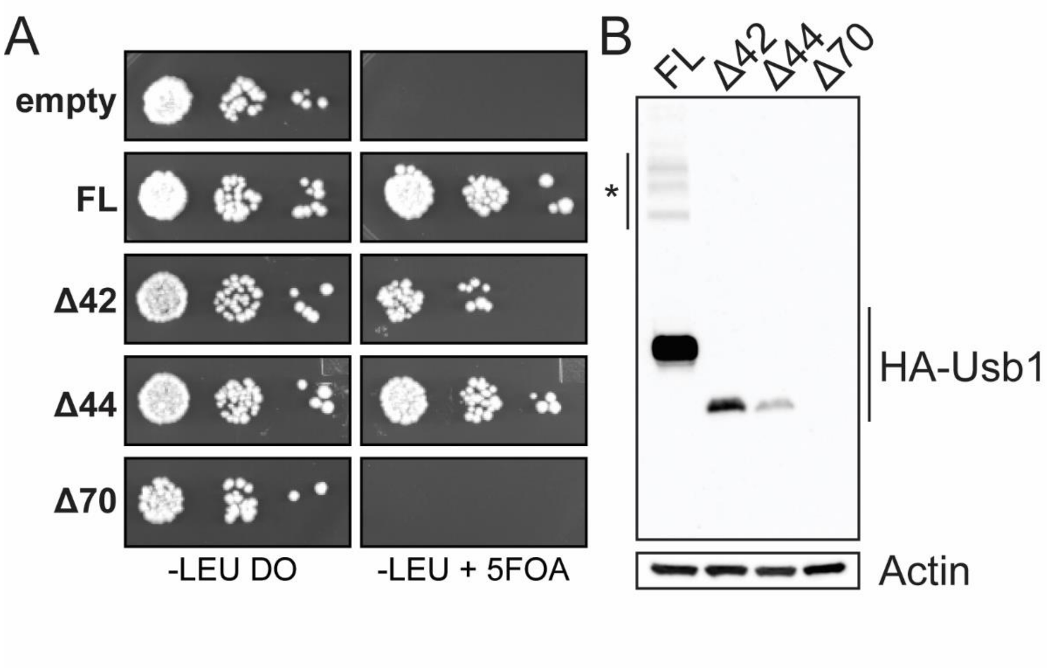

Supplementary Figure 5. The N-terminal domain is important for yUsb1 expression or stability.

(a) Serial dilutions of yeast containing full-length (FL) or N-terminally truncated (Δ42, Δ44, and Δ70) yUsb1 on a high-copy p425-GPD plasmid. yUsb1 is tagged with a single N-terminal HA tag.

(b) SDS-PAGE and western blotting analysis of Usb1 protein expression detected using a primary antibody to the genetically encoded N-terminal HA epitope tag. Actin was used as a loading control.

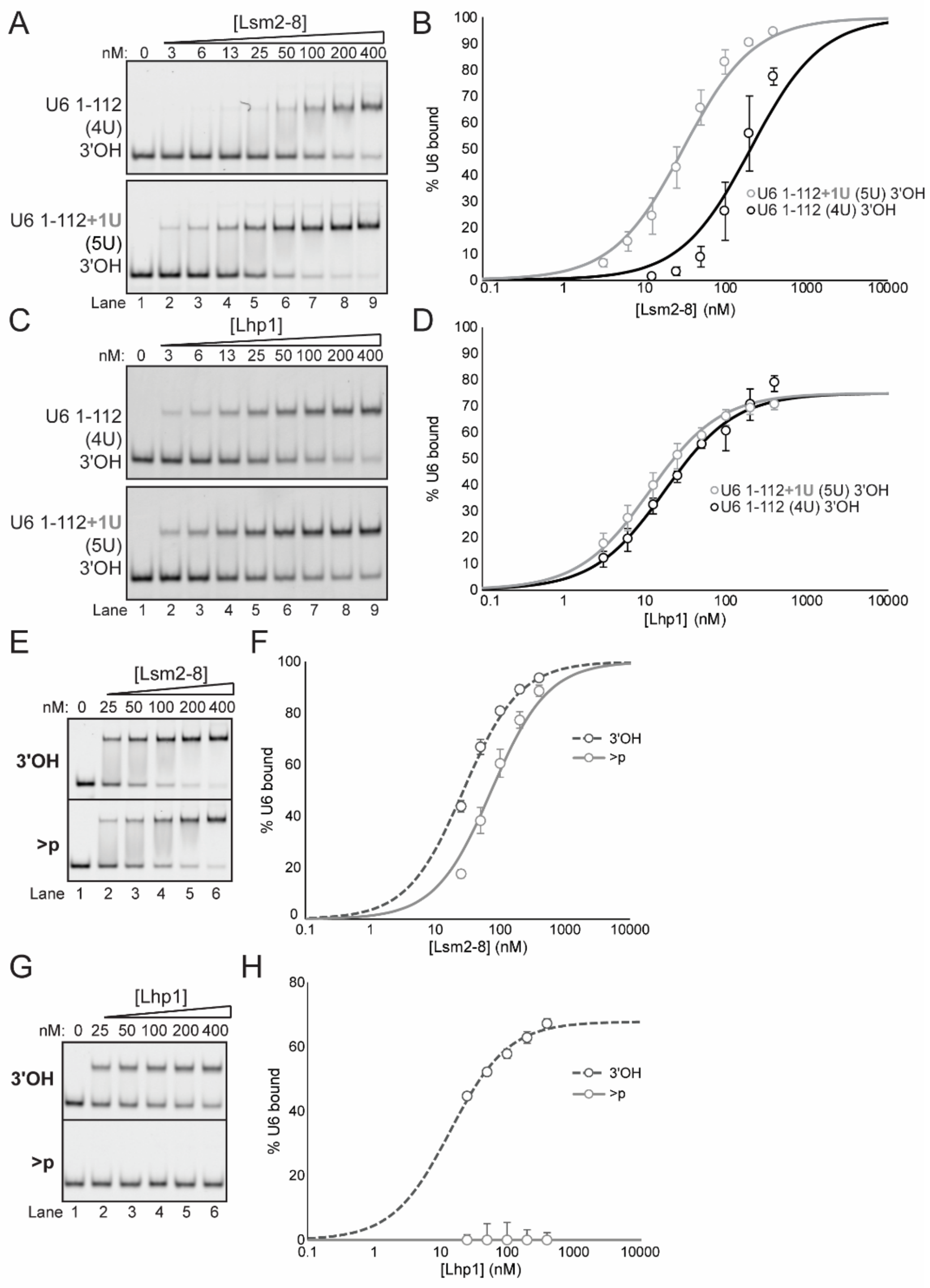
A
[Lsm2-8]
nM: 0 3 6 13 25 50 100 200 400
U6 1-112 (4U) 3'OH
U6 1-112+1U (5U) 3'OH
Lane 1 2 3 4 5 6 7 8 9
B
% U6 bound
[Lsm2-8] (nM)
U6 1-112+1U (5U) 3'OH
U6 1-112 (4U) 3'OH
C
[Lhp1]
nM: 0 3 6 13 25 50 100 200 400
U6 1-112 (4U) 3'OH
U6 1-112+1U (5U) 3'OH
Lane 1 2 3 4 5 6 7 8 9
D
% U6 bound
[Lhp1] (nM)
U6 1-112+1U (5U) 3'OH
U6 1-112 (4U) 3'OH
E
[Lsm2-8]
nM: 0 25 50 100 200 400
3'OH
>p
Lane 1 2 3 4 5 6
F
% U6 bound
[Lsm2-8] (nM)
3'OH
>p
G
[Lhp1]
nM: 0 25 50 100 200 400
3'OH
>p
Lane 1 2 3 4 5 6
H
% U6 bound
[Lhp1] (nM)
3'OH
>p

Supplementary Figure 6. Lsm2-8 preferentially binds full-length U6 RNA with an additional uridine.

(a) Native gel analysis comparing Lsm2-8 affinity to full-length fluorescently labeled U6 1-112 with a 2',3'-cis diol versus U6 1-112 with an additional uridine at the 3' end (U6 1-112+1U) and a 2',3'-cis diol. (b) Quantification of Lsm2-8 binding to U6 in (a). Plotted data points represent the average of three technical replicates ± s.d. (c) Native gel analysis comparing Lhp1 affinity to RNAs as described in (A). (d) Quantification of Lhp1 binding to U6 in (c). Plotted data points represent the average of three technical replicates ± s.d. (e) Native gel analysis comparing Lsm2-8 affinity to full-length fluorescently labeled U6 with an additional uridine at the 3' end (U6 1-112+1U) and different 3' modifications. Lsm2-8 binds U6 with a 2',3'-cis diol better than U6 with a 2',3'-cyclic phosphate. (f) Quantification of Lsm2-8 binding to U6 in (e). Plotted data points represent the average of three technical replicates ± s.d. (g) Native gel analysis comparing Lhp1 affinity to RNAs as described in (e). Lhp1 binds U6 with a 2',3'-cis diol, but not a 2',3'-cyclic phosphate. (h) Quantification of Lhp1 binding to U6 in (g). Plotted data points represent the average of three technical replicates ± s.d.

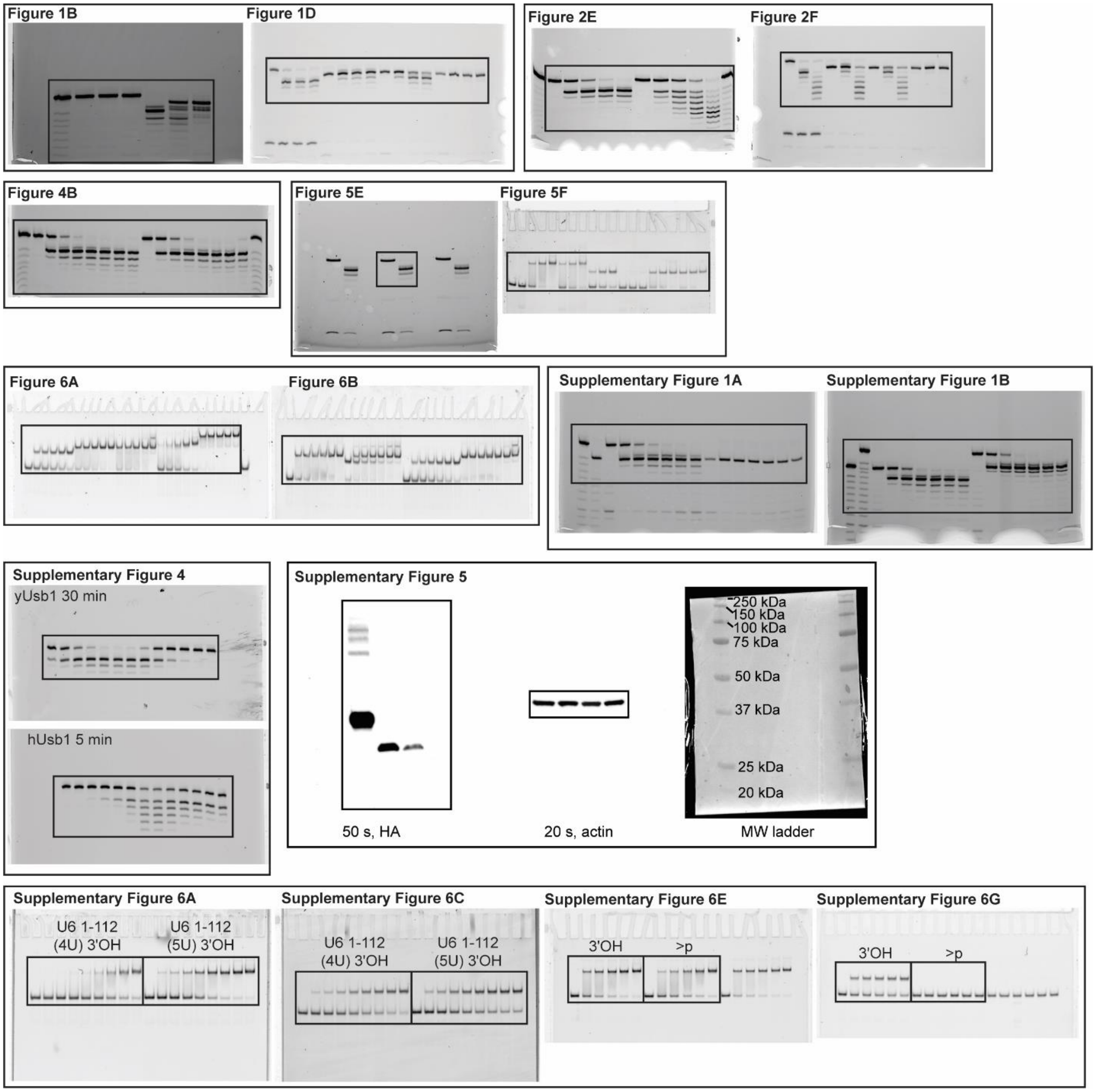

Supplementary Figure 7. Uncropped gel scans from Figures 1, 2, 5, 6, and Supplementary Figures 1, 4-6.

Supplementary Table 1. Summary of the effect of mutations and truncations of yUsb1 *in vivo*.

***USB1* allele**	***USB1Δ* complementation**
<u>*pRS414 backbone*</u>	
Vector	-
wild type	+++
Point mutations	
W75A	+++
F78A	+
F78H	+++
Y80A	+++
H133A*	-
H133N*	-
H231A*	-
H231N*	-
N-terminal truncations	
usb1Δ1-20	+++
usb1Δ1-41	+++
usb1Δ1-42	+++
usb1Δ1-43	+++
usb1Δ1-44	-
usb1Δ1-45	-
usb1Δ1-46	-
usb1Δ1-47	-
usb1Δ1-48	-
usb1Δ1-49	-
usb1Δ1-50	-
usb1Δ1-50 + SV40 NLS	-
usb1Δ1-70 + SV40 NLS	-

Mutations or truncations were introduced into pRS414-Usb1 and plated on media lacking TRP and containing 5-FOA. Ability to complement loss of the *URA3* marked wild-type Usb1 plasmid is listed in the right hand column, where "-" indicates no growth and "+++" indicates growth similar to wild-type Usb1. * indicates strains that were not tested by serial dilution.

Supplementary Table 2. DALI search of unique structural homologs to yUsb1 catalytic domain.

PDB ID	Z-score	rmsd	% identity	Name	Activity
4H7W	16.2	3.1	14	*Hs*Usb1	RNA exonuclease, 3'-5' phosphodiesterase, **leaves >p**
1VDX	12	3.9	13	*Ph*RNL	unknown, putative RNA 2'-5' ligase/2',3' CPDase
4QAK	12	3.3	11	*Ec*ThpR	2',3' CPDase, **leaves 2'P**
5JJ2	11.6	3.8	11	*Hs*AKAP18	binds NMPs, unknown activity, not a 3',5' CPDase
2VFL	11.5	4	10	*Rn*AKAP18	binds NMPs, unknown activity, not a 3',5' CPDase
1IUH	11.3	3.6	12	*Tt*RNL	unknown, putative RNA 2'-5' ligase/2',3' CPDase
2D4G	10.7	4.3	13	*Bs*YjcG	unknown, putative 2'-5' ligase/2',3' CPDase
2FYH	9.8	3.9	14	PF0027	putative RNA 2'-5' ligase/known 2',3' CPDase, **leaves 2'P**
4Z5V	9.4	3.9	16	MHV ns2	2'-5' phosphodiesterase (cleaves 5'AMP from 2' termini of 2-5A)
2FSQ	9	4.4	5	ATU0111	unknown
5AF2	8.7	3.3	11	VP3	2'-5' phosphodiesterase (cleaves 5'AMP from 2' termini of 2-5A)
1JH7	8.6	4.5	9	*At*CNPase	ADP-ribose 1'',2'' CPDase, leaves PO_3 on 1'' position
2YP0	5.6	3.8	9	*Mm*CNPase	2',3'-cyclic nucleotide 3'- phosphodiesterase, **leaves 2'P**
1WOJ	5.3	3.9	9	*Hs*CNPase	2',3'-cyclic nucleotide 3'- phosphodiesterase, **leaves 2'P**
2ILX	5.2	5.1	13	*Rn*CNPase	2',3'-cyclic nucleotide 3'- phosphodiesterase, **leaves 2'P**
2I3E	4.3	4.4	11	*Ca*RICH	2',3' CPDase, **leaves 2'P**

Supplementary Table 3. List of synthetic DNA oligonucleotides.

**Oligos to make pET28b-HT-yUsb1 1-290, truncation, and mutations**	
BamHI-pET28b-USB1	5'-CGGGATCCAATGGAATTCATATCTGCAGACTATTC-3'
XhoI-pET28b-USB1	5'-TGCTCGAGTTAGTTAAAAGGGATTCTAATGGAGTG-3'
Usb1-1-for	5'-ATGGAATTCATATCTGCAGACTATTCTAGCAGTGACGG-3'
Usb1-71-for	5'-ATGAGTCGATTTTGGCGTTCGTTCACATATTTTGAGTG GC-3'
His-TEV-rev	5'-GCCCTGGAAGTACAGGTTCTCGCTGCTGTGATGATGA TGATGATGGC-3'
Usb1_F78A_for	5'-GCGACATATTTTGAGTGGCGTCCGACTCCAGCG-3'
Usb1_F78H_for	5'-CATACATATTTTGAGTGGCGTCCGACTCCAGCG-3'
Usb1_F78_rev	5'-CGAACGCCAAAATCGACTCATATTCATATCC-3'
**Oligos to make p3HT-HT-hUsb1 79-265**	
hUsb1-D78N-for	5'-GGAAACTGGGCTACACATGTGTACGTTCCT-3'
hUsb1-TEV-rev	5'-CATATGAGCGCCCTGGAAATACAGGTTTTC-3'
**Oligos to make pET28b-Smt3-TEV-AtRNL(677-1104)**	
Trunc-AtRNL-for	5'-GCTAGCGAAAACCTGTATTTCCAGGGCGCTGTGAAGG AAGCTGTCCAAAAGGATGAG-3'
Trunc-AtRNL-rev	5'-GTGATGATGATGATGATGATGATGATGATGGCCCATGGT ATATCTCCTTCTTAAAGTTAA-3'
**Oligos to amplify *hph* gene flanked by Usb1 up and downstream region for *USB1* deletion strain:**	
Usb1_deletion_for	5'-CGAACCCAAACTATCTGTGACATCAGTTGCTGATGCTC TTCTTGGTCCCTTATTTGCGGTTTCAGAGACGTCCAGGC GCGCCAGATCTGTTTAGCTTGCCTTG-3'
Usb1_deletion_rev	5'-CGGGTGGGGGTATGGTAGGATTGACATTATCATGATG TTGAACATATCCTCATCTTGAGTGTCTTTCGCGAAGCGA GCTCGTTTTCGACACTGGATGGC-3'
Usb1_del_con_for	5'-GTTTCCTTAACAAAGGGAATCTC-3'
Usb1_del_con_rev	5'-TACCTAACAAGTTATCGAGG-3'
**Oligos to create pRS414-Usb1 and pRS416-Usb1:**	
BamHI-Usb1+500-for:	5'-ACGGGATCCATAGTTGCACACCCGCTACTCCCTCATT GACAGC-3'
XhoI-Usb1+500-rev	5'-CAGCTCGAGCTGAGGGATTTATATACCACGGATCTAC-3'
**Cloning oligos to introduce mutations into pRS414-Usb1:**	
Usb1_W75A_for	5'-GCGCGTTCGTTCACATATTTTGAGTGGCGTCCG-3'
Usb1_W75A_rev	5'-AAATCGACTCATATTCATATCCTGGTGCTCG-3'
Usb1_F78A_for	5'-GCGACATATTTTGAGTGGCGTCCGACTCCAGCG-3'
Usb1_F78H_for	5'-CATACATATTTTGAGTGGCGTCCGACTCCAGCG-3'
Usb1_F78_rev	5'-CGAACGCCAAAATCGACTCATATTCATATCC-3'
Usb1_Y80A_for	5'-GCTTTTGAGTGGCGTCCGACTCCAGCGATTC-3'
Usb1_Y80A_rev	5'-TGTGAACGAACGCCAAAATCGACTCATATTC-3'
Usb1_H133A_for	5'-GCGGTTTCCTTAACTCGATCGTTGTTGTTTGAAAC-3'
Usb1_H133N_for	5'-AACGTTTCCTTAACTCGATCGTTGTTGTTTGAAAC-3'
Usb1_H133_rev	5'-TAAAGGTTTGGGGGCTCCCAAGTGTGAGATGAAC-3'
Usb1_H231A_for	5'-GCGGTTTCAATTGCCATCGCTAGCAACCCTTCAAAAG-3'
Usb1_H231N_for	5'-AACGTTTCAATTGCCATCGCTAGCAACCCTTCAAAAG-3'
Usb1_H231_rev	5'-TAAATTTTGCCTACTTACAATTAAATCCTGATAG-3'

Usb1+500_START-rev	5'-CATAGGCCAGTCTTCTTCTAAGCGATTGGTAACC-3'
Usb1+500_SV40-rev	5'-AACTTTTCTTTTTTTTTTTGGTGGCATAGGCCAGTCTTCT TCTAAGCGATTGGTAACC-3'
Usb1-21_for	5'-AGTAACAAAAGTGAGGTCCAAATCGAATACACG-3'
Usb1-42_for	5'-ACTGATTTACCTGCAATACCGGATTCAATTATATTG AAG-3'
Usb1-43_for	5'-GATTTACCTGCAATACCGGATTCAATTATATTG-3'
Usb1-44_for	5'-TTACCTGCAATACCGGATTCAATTATATTGAAG-3'
Usb1-45_for	5'-CCTGCAATACCGGATTCAATTATATTGAAGTAC-3'
Usb1-46_for	5'-GCAATACCGGATTCAATTATATTGAAGTACCAC-3'
Usb1-47_for	5'-ATACCGGATTCAATTATATTGAAGTACCACATC-3'
Usb1-48_for	5'-CCGGATTCAATTATATTGAAGTACCACATCCCT-3'
Usb1-49_for	5'-GATTCAATTATATTGAAGTACCACATCCCTCCG-3'
Usb1-50_for	5'-TCAATTATATTGAAGTACCACATCCCTCCGAAT-3'
Usb1-51_for	5'-ATTATATTGAAGTACCACATCCCTCCGAATTTAC-3'
Usb1-71_for	5'-ATGAGTCGATTTTGGCGTTCGTTCACATATTTTGAGT GGC-3'
**Oligos to make p425-GPD-yUsb1 and hUsb1, truncations, and mutations**	
BamHI-yUsb1-for	5'-CGGGATCCAATGGAATTCATATCTGCAGACTATTC-3'
SalI-yUsb1-rev	5'-GGCGTCGACTTAGTTAAAAGGGATTCTAATGGAGTGA-3'
BamHI-hUsb1-for	5'-ACGGGATCCATATGAGTGCAGCCCCTTTAGTAGGG-3'
SalI-hUsb1-rev	5'-GGCGTCGACTCATTTCAGCGGCATGGAAAAAAATTTG-3'
p425-up-HA-r	5'-AGCGTAATCTGGTACGTCGTATGGGTACATATGGAT CCACTAGTTCTAGAATCCGTCG-3'
Usb1-1-for	5'-ATGGAATTCATATCTGCAGACTATTCTAGCAGTGACGG-3'
Usb1-43_for	5'-GATTTACCTGCAATACCGGATTCAATTATATTG-3'
Usb1-45_for	5'-CCTGCAATACCGGATTCAATTATATTGAAGTAC-3'
Usb1-71_for	5'-ATGAGTCGATTTTGGCGTTCGTTCACATATTTTGAGT GGC-3'
hUsb1-H84F-for	5'-TTCGTCTACGTCCCGTATGAAGCCAAGGAGGAA-3'
hUsb1-H84-rev	5'-AGTTGCCCAGTTCCCACGCTCGTGAGGAAA-3'
hUsb1-wt-79-for	5'-GGGAACTGGGCAACTCATGTCTACGTCCCG-3'
hUsb1-H84F-79-for	5'-GGGAACTGGGCAACTTTCGTCTACGTCCCG-3'
**Oligos to make HH-U6 95-112+3U-HDV plasmid**	
HH-U6_95-112(UUU)-for	5'-AAACACGTTCGCGTGTCAGAGATTTATTTCGTTTTTTG GGTCGGCATGGCATCTCCACCTCCTCGCGGTCCG-3'
HH-U6_95-112(UUU)-rev	5'-CGGCCTAACGGCCTCATCAGAGAGATTTATTCTCCCTAT AGTGAGTCGTATTAGAAGAGC-3'
**Oligos to make HH-U6 13-112-HDV plasmid**	
U6_13-112_for	5'-CGAAACACGTTCGCGTGTCACCCTTCGTGGACATTTGGT CAATTTGAAACAATACAGAGA-3'
HH-U6_13-112_rev	5'-GCCTAACGGCCTCATCAGACCCTTCGTGGCTCCCTATAGT GAGTCGTATTAAAG-3'

Supplementary Table 4. Nucleotide sequence of synthetic genes.

hUsb1, codon optimized for *S. cerevisiae* (used for *in vivo* complementation assays)	5'-GGATCCATATGAGTGCAGCCCCTTTAGTAGGGTACAGCTCAA<br>GCGGGAGTGAAGATGAGAGCGAGGACGGAATGAGGACCAGACCGG<br>GTGATGGAAGTCATAGAAGAGGCCAGAGCCCCTTGCCGAGGCAAC<br>GTTTCCCCGTACCCGACAGTGTTTTGAATATGTTCCCAGGTACCGAA<br>GAGGGACCCGAAGATGACTCTACCAAACACGGAGGGAGGGTCCGT<br>ACTTTTCCTCACGAGCGTGGGAACTGGGCAACTCATGTCTACGTCC<br>CGTATGAAGCCAAGGAGGAATTTTTGGATCTGTTGGATGTGCTACTT<br>CCCCACGCCCAAACCTACGTACCGAGACTGGTAAGGATGAAAGTCT<br>TCCACCTGAGTCTGAGTCAGAGCGTTGTGCTGAGGCATCACTGGAT<br>ACTGCCTTTCGTACAGGCGCTTAAGGCTCGTATGACCAGTTTTCACA<br>GGTTCTTTTTTACTGCCAATCAGGTGAAGATATATACAAACCAAGAAA<br>AAACAAGGACCTTTATTGGCCTAGAGGTTACTTCCGGTCACGCACAA<br>TTTTTGGACCTAGTCTCCGAGGTTGACCGTGTGATGGAAGAATTTAA<br>CCTGACCACTTTTTATCAAGACCCTTCATTCCATCTGTCACTTGCATG<br>GTGTGTCGGAGATGCTCGTCTGCAACTAGAAGGGCAATGTCTTCAA<br>GAGCTGCAAGCCATAGTCGATGGTTTCGAGGATGCTGAGGTCCTGC<br>TTAGAGTCCACACAGAGCAGGTGCGTTGCAAGAGTGGCAACAAATT<br>TTTTTCCATGCCGCTGAAATGAGTCGAC-3'
hUsb1, codon optimized for *E. coli* (used for protein expression and crystallization)	5'-CATATGTCAGCTGCACCATTAGTCGGGTATTCATCATCGGGTTC<br>TGAGGATGAGTCCGAGGACGGGATGCGTACACGTCCTGGCGACGG<br>ATCGCACCGTCGTGGGCAGTCCCCATTGCCTCGCCAGCGCTTCCCT<br>GTGCCCGATTCGGTCCTGAACATGTTTCCAGGGACCGAGGAAGGAC<br>CGGAAGATGATAGCACTAAGCATGGAGGTCGCGTACGCACTTTCCC<br>ACACGAGCGCGGAAACTGGGCTACACATGTGTACGTTCCTTACGAA<br>GCGAAGGAAGAATTCCTGGATTTGCTTGATGTCTTGCTTCCACATGC<br>GCAGACTTACGTGCCGCGCTTAGTCCGCATGAAAGTCTTTCACCTG<br>TCCTTGTCCCAATCGGTAGTTCTTCGCCATCATTGGATTTTGCCTTTT<br>GTGCAGGCGTTGAAGGCCCGTATGACATCGTTCCACCGCTTTTTTT<br>CACCGCCAATCAGGTAAAGATTTATACAAATCAAGAGAAGACACGCA<br>CATTCATTGGCTTGGAGGTAACCTCAGGGCACGCTCAATTTTTGGAC<br>TTAGTAAGTGAAGTAGACCGCGTGATGGAGGAGTTCAACCTTACAA<br>CTTTCTATCAAGATCCGTCCTTCCACCTGAGCTTAGCCTGGTGCGTG<br>GGAGATGCTCGCCTTCAGTTGGAAGGCCAATGCCTTCAAGAACTGC<br>AAGCAATCGTAGACGGGTTCGAGGACGCCGAGGTACTGCTTCGTGT<br>ACACACCGAGCAGGTTCGCTGCAAATCAGGTAATAAGTTTTTCTCCA<br>TGCCACTTAAATGACTCGAGGATCC-3'
Lhp1 from *S. cerevisiae*, codon optimized for *E. coli* (used for protein expression)	5'-CATATGTCTGAAAAACCGCAGCAGGAAGAACAGGAAAAACCGC<br>AGTCTCGTCGTAACTCTTTCGCTGTTATCGAATTCACCCCGGAAGTT<br>CTGGACCGTTGCCTGAAACAGGTTGAATTCTACTTCTCTGAATTCAA<br>CTTCCCGTACGACCGTTTCCTGCGTACCACCGCTGAAAAAAACGAC<br>GGTTGGGTTCCGATCTCTACCATCGCTACCTTCAACCGTATGAAAAA<br>ATACCGTCCGGTTGACAAAGTTATCGAAGCTCTGCGTTCTTCTGAAA<br>TCCTGGAAGTTTCTGCTGACGGTGAAAACGTTAAACGTCGTGTTCCG<br>CTGGACCTGACCGCTGCTCGTAACGCTCGTATCGAACAGAACCAGC<br>GTACCCTGGCTGTTATGAACTTCCCGCACGAAGACGTTGAAGCTTCT<br>CAGATCCCGGAACTGCAGGAAAACCTGGAAGCTTTCTTCAAAAAACT<br>GGGTGAAATCAACCAGGTTCGTCTGCGTCGTGACCACCGTAACAAA<br>AAATTCAACGGTACCGTTCTGGTTGAATTCAAAACCATCCCGGAATG<br>CGAAGCTTTCCTGAAATCTTACTCTAACGACGACGAATCTAACGAAA

	TCCTGTCTTACGAAGGTAAAAAACTGTCTGTTCTGACCAAAAAACAG TTCGACCTGCAGCGTGAAGCTTCTAAATCTAAAAACTTCTCTGGTCG TTCTCGTTCTTTCAACGGTCACAAAAAAAAAAAACCTGCCGAAATTCC CGAAAAACAAAAAAAAAAACGGTAAAGAAGAATCTAAAGAAGACTCT TCTGCTATCGCTGACGACGACGAAGAACACAAAGAATGATGACTCG AGGATCC-3'

Supplementary Table 5. List of synthetic RNA oligonucleotides.

FAM-U6 95-112	5'-/56-FAM/rArGrArGrArUrUrUrArUrUrUrCrGrUrU rUrU-3'
FAM-U6 95-112-3P	5'-/56-FAM/rArGrArGrArUrUrUrArUrUrUrCrGrUrU rUrU/3Phos/-3'
FAM-U6 95-112+1U	5'-/56-FAM/rArGrArGrArUrUrUrArUrUrUrCrGrUrU rUrUrU-3'
FAM-U6 95-112+1U-3P	5'-/56-FAM/rArGrArGrArUrUrUrArUrUrUrCrGrUrU rUrUrU/3Phos/-3'
FAM-U6 95-112+3U	5'-/56-FAM/rArGrArGrArUrUrUrArUrUrUrCrGrUrU rUrUrUrUrU-3'
FAM-U6 95-112+3U-3P	5'-/56-FAM/rArGrArGrArUrUrUrArUrUrUrCrGrUrU rUrUrUrUrU/3Phos/-3'
FAM-U6 95-112+6U	5'-/56-FAM/rArGrArGrArUrUrUrArUrUrUrCrGrUrU rUrUrUrUrUrUrUrU-3'
FAM-U6 95-111dUrU	5'-/56-FAM/rArGrArGrArUrUrUrArUrUrUrCrGrUrU rU/ideoxyU/rU-3'
FAM-U6 84-94	5'-/56-FAM/rCrCrGrUrUrUrUrArCrArA-3'
FAM-U6 84-112+3U-3P	5'-/56-FAM/rCrCrGrUrUrUrUrArCrArArArGrArGrA rUrUrUrArUrUrUrCrGrUrUrUrUrUrU/3Phos/-3'
5'Cy3-U6 1-12	5'-/5Cy3/rGrUrUrCrGrCrGrArArGrUrA-3'

SUPPLEMENTARY REFERENCES

1. Hilcenko, C. *et al.* Aberrant 3' oligoadenylation of spliceosomal U6 small nuclear RNA in poikiloderma with neutropenia. *Blood* **121**, 1028-1038 (2013).
